# The Panther Fusion System with Open Access Functionality for Laboratory-Developed Tests for Influenza A Virus Subtyping

**DOI:** 10.1128/JCM.00188-20

**Published:** 2020-05-26

**Authors:** Kathleen A. Stellrecht, Jesse L. Cimino, Vincente P. Maceira

**Affiliations:** a Department of Pathology and Laboratory Medicine, Albany Medical Center Hospital, Albany, New York, USA; b Department of Pathology and Laboratory Medicine, Albany Medical College, Albany, New York, USA; c Department of Immunology and Microbial Diseases, Albany Medical College, Albany, New York, USA; UNC School of Medicine

**Keywords:** influenza A virus typing, PCR, Panther Fusion, Open Access, laboratory-developed tests (LDTs), influenza

## Abstract

Nucleic acid amplification tests, such as PCR, are the method of choice for respiratory virus testing, due to their superior diagnostic accuracy and fast turnaround time. The Panther Fusion (Fusion; Hologic) system has an array of highly sensitive *in vitro* diagnostic (IVD) real-time PCR assays for respiratory viruses, including an assay for influenza A (FluA) virus, influenza B (FluB) virus, and respiratory syncytial virus (RSV) (FFABR assay). The Fusion system has Open Access functionality to perform laboratory-developed tests (LDTs) alongside IVD assays. We developed two LDTs for FluA virus strain typing on the Panther Fusion instrument, enabling side-by-side testing with the FFABR assay. The LDT-FAST assay uses proprietary primers and probes designed by Hologic for the Prodesse ProFAST+ (PFAST) assay. The exWHO-FAST assay is an expanded redesign of the WHO-recommended reverse transcriptase PCRs (RT-PCRs). To evaluate the performance of these two LDTs, 110 FluA virus-positive samples were tested. Of these, 104 had been subtyped previously; 54 were H3, 46 were 09H1, and 4 were fsH1. All were appropriately subtyped by both LDTs. Of the untyped FluA virus samples, three were subtyped as H3 by both LDTs and two were subtyped as H3 by the LDT-FAST assay only. The sample not subtyped by either LDT was retested with the FFABR assay and was now negative. Limit-of-detection (LOD) analyses were performed with five FluA virus strains. The LDT-FAST LODs were similar to the FFABR assay LODs, while the exWHO-FAST LODs were higher for two H3N2 strains, findings that were explained by analysis of primer/probe homology. In conclusion, either FluA virus typing assay would be a valuable complement to the Panther Fusion respiratory menu given the performance of these LDTs, the system’s full automation, and the ability to split eluates for both IVD and LDT testing.

## Abstract (precis)

Nucleic acid amplification tests, such as PCR, are the method of choice for respiratory virus testing, due to their superior diagnostic accuracy and fast turnaround time. The Panther Fusion (Fusion; Hologic) system has an array of highly sensitive *in vitro* diagnostic (IVD) real-time PCR assays for respiratory viruses, including an assay for influenza A (FluA) virus, influenza B (FluB) virus, and respiratory syncytial virus (RSV) (FFABR assay). The Fusion system has Open Access functionality to perform laboratory-developed tests (LDTs) alongside IVD assays.

## INTRODUCTION

Influenza viruses are classified into three distinct types, A, B, and C, with subdivisions in type A based on antigenic characterization of the surface glycoproteins. Currently circulating strains of influenza A (FluA) virus include H3N2 and H1N1pdm09. Since the 2009 influenza pandemic, H1N1pdm09 quickly became the dominant H1 strain, displacing the former seasonal H1N1 (fsH1) strain; however, rare occurrences of fsH1 have been observed. This is fortunate, because the fsH1 strain had become universally resistant to oseltamivir (Tamiflu). The clinical significance of the H3N2 strain is its association with higher attack rates, hospitalization rates (1), and mortality (2, 3), as well as the suboptimal vaccine efficacy against this strain (4). Because coinfection with multiple strains can occur, influenza strain typing is important for proper assignment of patients to cohorts. Furthermore, FluA virus subtyping not only has therapeutic and clinical benefits; it is an important epidemiological tool with public health benefits.

Panther Fusion (referred to below as Fusion) is a fully automated high-throughput system with on-demand testing capabilities. This system integrates nucleic acid extraction with either transcription-mediated amplification (TMA) or real-time PCR. The *in vitro* diagnostic (IVD) respiratory virus assays on the Fusion instrument, which include the FluA–FluB–respiratory syncytial virus (FluA/B/RSV [(FFABR]), Paraflu, and adenovirus–human metapneumovirus–rhinovirus (AdV/hMPV/RV) assays, have been shown to have exquisite sensitivity (5). Furthermore, the Fusion system has Open Access functionality, which enables the performance of laboratory-developed tests (LDTs) with full automation. Not only can LDTs be run alongside IVD tests; they can be run from the same nucleic acid extract. Our laboratory developed and compared two LDTs on the Fusion instrument for the subtyping of FluA virus, to complement the respiratory virus assays. The results were compared to those of the FFABR assay for sensitivity in viral detection and to those of the Prodesse ProFAST+ (PFAST) assay for subtyping accuracy. Furthermore, the clinical specimens used were analyzed previously by the following methods, in addition to the FFABR and PFAST assays: the cobas Influenza A/B (cIAB) test, FilmArray Respiratory Panel 1.7 (RP), and the Xpert Flu (Xpt) assay (5, 6). The PFAST and RP assays provided subtype analysis for H3, 09H1, and fsH1 strains, while the Xpt assay provided a 09H1 call-out result. This sample set provided insight into the sensitivities and accuracies of the LDTs relative to those of commercial assays.

(This study was presented in part at both the 2019 ASM Clinical Virology Symposium and ASM Microbe 2019.)

## MATERIALS AND METHODS

### Specimens.

Clinical specimens comprised 110 nasopharyngeal swab (NP) specimens, in 3 ml of viral transport medium (VTM), previously determined to be positive for FluA virus by the FFABR assay (Hologic, San Diego, CA). FluA virus subtypes had been determined by the PFAST assay (Hologic) for 104 specimens. These samples were used in previous studies comparing influenza virus PCR assays (5, 6) and were also analyzed by the following methods: the cIAB test (Roche Diagnostics, Indianapolis, IN), RP (BioFire, Salt Lake City, UT), and the Xpt assay (Cepheid, Carlsbad, CA). Of the six nontyped FluA virus specimens, two had been FluA virus positive by both the cIAB and FFABR tests and four by the FFABR assay only. Specimens were stored at –80°C but had undergone multiple freeze-thaw cycles.

### Viruses.

Three FluA virus isolates obtained from the ATCC (Manassas, VA) included an fsH1 strain, A/Florida/3/2006 (VR-1893); an H1N1pdm09 (09H1) strain, A/California/7/2009(H1N1)pdm09 (VR-1894); and an H3N2 clade 3C.3a isolate, A/Switzerland/9715293/2013 (Swiss; VR-1837). An H3N2 clade 3C.1 isolate, A/Texas/50/2012(H3N2)-like (Texas), was obtained through the New York State Department of Health (NYS DOH) Proficiency Testing program. A variant H3N2 strain, A/Indiana/09/2012(H3N2v)-like, was a gift from Judith Lovchik, Indiana State Department of Health. Viral stocks were serially diluted 1:10 in VTM and the dilutions tested six to eight times for limit-of-detection (LOD) and precision analyses. The 1:10 dilutions for the fsH1, 09H1, and Swiss strains were also diluted 1:4 to meet assay validation requirements in New York State. These dilutions were tested simultaneously by the LDT-FAST, exWHO-FAST, and FFABR assays, using the same nucleic acid extract. Determinations of viral nucleic acid concentrations were based on quantified control viral RNA (Hologic, Inc.) using either the Prodesse ProFlu+ (Hologic) or the PFAST assay, depending on strain/clade-based amplification efficiency (6). The Swiss, 09H1, and fsH1 strains were combined at concentrations of 3.77, 4.83, and 3.81 log_10_ copies/ml, respectively, for use as a positive control and for additional precision and reagent stability studies.

### Test methods.

The test methods investigated in this study are listed in Table 1.

**TABLE 1:** Test methods used in this study

Abbreviation	Description
Fusion	Panther Fusion (instrument)
LDT-FAST	Laboratory-developed test for influenza virus typing using proprietary primers and probes designed by Hologic for the Prodesse ProFAST+ assay
exWHO-FAST	Laboratory-developed test for influenza virus typing using WHO-recommended primers and probe, modified for optimal performance on the Fusion instrument and expanded for the inclusion of former seasonal H1N1 viruses
FFABR	Panther Fusion FluA/B/RSV assay for the detection of influenza A virus, influenza B virus, and respiratory syncytial virus
PFAST	Prodesse ProFAST+ assay for influenza A virus strain typing
Xpt	Xpert Flu assay for the detection of influenza viruses A and B, with an H1 call-out
RP	FilmArray Respiratory Panel 1.7
cIAB	cobas Influenza A/B test

### Nucleic acid extraction and amplification.

The LDTs were performed on the fully automated Panther Fusion system with Open Access functionality. This instrument utilizes universal nucleic acid extraction reagents and adds internal control (IC) RNA at the initiation of extraction. Following the standard protocol for respiratory virus detection, 500 μl of specimen was added to a Panther Fusion specimen lysis tube containing 750 μl buffer and 360 μl of the mixture used for extraction. The nucleic acid was subsequently eluted into 50 μl, and 5 μl was amplified for an effective sample volume of 14.4 μl.

High-performance liquid chromatography (HPLC)-purified primers and probes for the LDT-FAST test (Biosearch Technologies, Petaluma, CA) were combined with 8 mM Tris (pH 8.0; Hologic), 4 mM MgCl_2_ (Hologic), 50 mM KCl (Hologic), and RNA-IC primers and probes (Hologic), and the mixture was overlaid with Panther Fusion oil reagent (Hologic). exWHO-FAST (Integrated DNA Technologies, Inc., Coralville, IA) (Table 2) primers and probes were combined with the same concentrations of Tris and KCl and with 4.5 mM MgCl_2_, and the mixture was overlaid with oil. The Fusion system rehydrates the enzyme and nucleotide pellet with the primer, probe, and buffer mixture and adds it to the nucleic acid extract in 25-μl reaction mixtures. Real-time reverse transcriptase PCR (RT-PCR) was performed using the following amplification profile: 1 cycle of 8 min at 46°C, 1 cycle of 2 min at 95°C, and 45 cycles of 95°C for 5 s, with 60°C for 22 s.

**TABLE 2:** exWHO-FAST FluA virus typing primer/probe design

Target and primer or probe^*a*^	Sequence^*b*^	Concn (μM)	*T_m_* (°C)	Source or reference
H3				
H3-266′-F	ACC CTCAGTGTGATGGCTTTCAAA	0.65	64.0	7
H3-373′-R	TAAGGG AGGCATAATCCGGCACAT	0.65	64.8	
H3-315′-P	FAM-ACG AAGCAAAGC/ZEN/CTACAGCAACTGTT-BHQ1	0.8	68.2	
09H1				
swlH1F-786	GA CAAAATAACATTCGAAGCAACTGG	0.75	64.1	7
swlH1R-939	GGGA GGCTGGTGTTTATAGCACC	0.75	63.5	
swlH1-832P	Cy5.5-G CATTCGCAATGGAAAGAAATGCTGG-BHQ3	0.6	67.7	
fsH1				
fsH1-169F	AAACTRTGTCTATTAAAAGGAAWAGCC	0.85	64.1	Our own design
fsH1-297R	GTYTCTCTACAATGTAKGACCA	0.85	62.3	
fsH1-244P	TxR-CCCAGAATGCRAATTACTGATTTCC-BHQ2	0.6	65.9	

a F, forward primer; R, reverse primer; P, probe.

b Modifications from the WHO design are indicated as follows: nucleotides in strikethrough font were removed from the WHO-designed sequences, and underlined nucleotides were modified for better coverage of circulating strains of virus. FAM, 6-carboxyfluorescein; BHQ, black hole quencher.

The primers and probes from both the WHO’s one-step duplex real-time RT-PCR (Annex 2B, Protocol 2) and its one-step real-time RT-PCR for the H1 gene of influenza A(H1N1)pdm09 virus (Annex 2B, Protocol 1) (7) were modified to have melting temperatures (*T_m_*) more compatible with the Fusion system’s optimal amplification profile. Based on assay optimization studies, it was determined that the the modified H3 primers and probe from WHO Protocol 2 and the modified H1N1pdm09 primers and probe from WHO Protocol 1 demonstrated superior performance in combination (data not shown). This multiplex was expanded into a triplex to include a target for the former seasonal H1 (fsH1) strain for a complete FluA virus strain-typing assay.

### HA sequence analysis.

For *in silico* analysis of primer and probe homology, hemagglutinin (HA) gene sequences were obtained from the Global Initiative on Sharing Avian Influenza Data (GISAID) EpiFlu database. This database comprises influenza sequences for the semiannual vaccine strain selection uniquely submitted from contributors such as the World Organisation for Animal Health (formerly the Office International des Epizooties), national reference laboratories, and all WHO Collaborating Centers for Surveillance, Epidemiology, and Control of Influenza (13). Included were all unique human isolate sequence data from the United States available as of 28 June 2019 but collected during the following periods: H3 and 09H1 isolates from 1 September through 30 April 2019, sH1 isolates from 1 September 2008 through 30 April 2009, and H3N2v isolates from 1 September 2017 through 31 October 2017 plus seven prototype strains (A/Kansas/13/2009, A/Pennsylvania/14/2010, A/Minnesota/11/2010, A/Indiana/08/2011, A/Indiana/10/2011, A/Iowa/07/2011, A/West Virginia/06/2011). Sequence alignments and motif searches were performed with MEGA, version 10.0.5 (Biodesign Institute, Tempe, AZ). Clade designations were based on signature amino acids (8).

### Statistical analysis.

Sensitivities and confidence intervals (CI) were determined using Microsoft Excel 2016 (Microsoft, Redmond, WA) (9). Probit analyses for the LOD with a 95% probability of detection were performed using SPSS, version 13.0 (IBM, Armonk, NY).

## RESULTS

Of the 104 previously subtyped FluA virus-positive clinical samples, 54 were H3, 46 were 09H1, and 4 were fsH1. All samples were appropriately subtyped by both the LDT-FAST and the exWHO-FAST assay (Table 3). Of the six previously untyped samples, three were subtyped as H3 by both assays. Another two were subtyped by the LDT-FAST test only as H3, and one sample was not subtyped by any method. The original threshold cycle (*C_T_*) values with the FFABR assay were 37.1 and 39.7 for the two samples subtyped by the LDT-FAST test only. The original FFABR assay *C_T_* value for the sample not subtyped by either LDT was 37.9. This sample was retested with the FFABR assay and was found negative upon repeat testing. Although these samples were not previously subtyped, they were all collected during a 2-week period in 2015 when the incidence of FluA virus infection was 25% and the subtype was exclusively H3 (5, 6). Based on this information, the sensitivities were 100% (95% CI, 100%) and 98.2% (95% CI, 95.6% to 100.7%) for the LDT-FAST and exWHO-FAST assays, respectively. Furthermore, these assays demonstrated higher sensitivity than the cIAB, PFAST, RP, and Xpt assays based on results from previous studies with the same samples (6).

**TABLE 3:** LDT-FAST assay performance with clinical specimens, compared with historical data

No. of specimens	Virus	Result^*a*^ by the following assay:						
		LDT-FAST	exWHO-FAST	FFABR	cIAB	PFAST	RP	Xpt
43	H3	H3+	H3+	+	+	H3+	H3+	+^*b*^
4	H3	H3+	H3+	+	+	H3+	H3+	–
3	H3	H3+	H3+	+	–	H3+	+^*c*^	–
1	H3	H3+	H3+	+	–	H3+	–	+^*b*^
1	H3	H3+	H3+	+	–	H3+	–	–
2	H3	H3+	H3+	+	+	–	–	–
3	FluA	H3+	H3+	+	–	–	–	–
2	FluA	H3+	–	+	–	–	–	–
1	+/–^*d*^	–	–	+/–	–	–	–	–
37	09H1	09H1+	09H1+	+	+	09H1+	H3+	09H1+
6	09H1	09H1	09H1			09H1+	+^*e*^	09H1+
2	09H1	09H1+	09H1+	+	+	09H1+	–	09H1+
1	09H1	09H1+	09H1+	+	+	09H1+	+^*f*^	–
4	fsH1^*g*^	fsH1+	fsH1+	+	+	fsH1+	fsH1+	+^*b*^

a +, positive; –, negative.

b Positive, not 09H1.

c Two isolates positive for FluA virus by RP but not typed, and one equivocal for FluA virus by RP.

d Repeat testing by the FFABR assay on an aliquot used for LDTs was negative.

e Six isolates equivocal for FluA virus by RP.

f Positive for FluA virus by RP, but not typed.

g fsH1, former seasonal H1N1.

The LODs for the LDT-FAST assay were comparable to those for the FFABR assay with all FluA virus isolates tested, including the currently circulating 09H1 isolate and the H3 clade 3C.3a isolates. The LODs for the exWHO-FAST assay were comparable to those for the FFABR and LDT-FAST assays with the 09H1, sH3, and H3 clade 3C.1 isolates but were higher with the clade 3C.3a and H3N2v isolates (Table 4). The LOD studies were also designed to demonstrate the intra- and interassay reproducibilities; three different concentrations of the samples were tested multiple times in one run of testing and were then retested on different days. The coefficients of variation (CV) for the intra-assay and interassay *C_T_* values, for individual targets, across three concentrations of virus, ranged from 0.8% to 2.7% with the LDT-FAST assay and from 0.7% to 2.7% with the exWHO-FAST assay (Table 5). Similarly, for the positive control in which targets are combined, the CV ranged from 2.7 to 2.9% for the LDT-FAST assay and from 0.8 to 1.0% for the exWHO-FAST assay over 20 days of testing. Since the same master mix preparations were on board the Panther Fusion instrument and were used for the duration of the 20-day reproducibility study, this analysis also demonstrated that the master mixes were stable onboard for at least 20 days.

**TABLE 4:** Limit-of-detection analyses with a selection of influenza virus strains

Virus	Strain	H3N2 clade^*a*^	LOD (log copies/ml) for the following assay:		
			LDT-FAST	exWHO-FAST	FFABR
09H1N1	A/California/07/2009-like	N.A.	3.16	3.16	3.06
fsH1N1	A/Florida/3/2006	N.A.	2.97	3.12	2.49
H3N2	A/Texas/50/2012-like^*b*^	3C.1	2.97	2.88	2.97
H3N2	A/Switzerland/9715293/2013	3C.3a	2.93	3.41	3.08
H3N2v^*c*^	A/Indiana/09/2012-like	H3N2v	3.04	3.37	3.08

a N.A., not applicable.

b Chimeric isolate with an A/Texas/50/2012-like HA gene and an A/Hong Kong/5738/2014-like M1 gene.

c A swine FluA virus strain that has been associated with human infection, particularly after exposure at petting zoos and agricultural fairs.

**TABLE 5:** Coefficients of variation for intra- and interassay reproducibility

Virus	Concn (log_10_ copies/ml)	Coefficient of variation (%) for the following assay:			
		LDT-FAST		exWHO-FAST	
		Intra-assay	Interassay^*a*^	Intra-assay	Interassay^*a*^
H3	4.50	2.2	2.3	1.5	2.5
	4.10	1.8	2.2	1.9	2.3
	3.10	2.5	2.7	2.1	2.1
09H1	5.64	0.9	0.8	2.6	2.7
	5.04	1.0	0.9	1.5	1.9
	4.04	2.7	2.3	2.2	2.3
fsH1	4.81	0.8	1.1	0.7	0.8
	4.21	1.0	1.4	0.7	1.0
	3.21	1.8	2.0	2.2	1.9

a Over three days of testing.

Because LOD performance appeared to be strain dependent, primer/probe homology was analyzed against circulating strains. Unique isolates from the United States uploaded to GISAID were aligned and the number of mismatches counted for each primer and probe. More than 97% of circulating H3 isolates demonstrated exact homology with the LDT-FAST primers and probes, and there was not a significant difference upon analysis by clade (Table 6). However, the exWHO-FAST assay demonstrated a number of mismatches that were clade associated. H3 primer/probe sets for both assays demonstrated mismatches with the H3N2v strains, as expected, since these targets were not designed to detect these isolates.

**TABLE 6:** Percentages of circulating FluA virus isolates demonstrating 0 to 3 mismatches to the FAST primers and probes^*a*^

Virus subtype	LDT-FAST assay					exWHO-FAST assay				
	No. of isolates	% of isolates with the following no. of base mismatches:				No. of isolates	% of isolates with the following no. of base mismatches:			
		0	1	2	3		0	1	2	3
H3 (2018–19)										
H3 forward primer	1,028	97.6	2.4	0	0	1,028	77.5	21.1	1.3	0.1
Clade 3C.2a	282	98.9	1.1	0	0	282	28.7	66.3	4.6	0.4
Clade 3C.3a	746	97.1	2.9	0	0	746	96.0	4.0	0.0	0.0
H3 reverse primer	1,028	98.4	1.6	0	0	1,028	23.0	75.7	1.4	0
Clade 3C.2a	282	95.4	4.6	0	0	282	80.9	18.4	0.7	0
Clade 3C.3a	746	99.6	0.4	0	0	746	1.1	97.3	1.6	0
H3 probe	1,028	97.9	2.0	0.1	0	1,028	5.9	32.1	61.4	0.6
Clade 3C.2a	282	96.8	2.8	0.4	0	282	21.6	69.1	8.9	0.4
Clade 3C.3a	746	98.3	1.7	0	0	746	0.0	18.1	81.2	0.7
H3N2v (2017)	39 (plus 5 prototype strains)					39 (plus 7 prototype strains)				
H3 forward primer		13.6	0	86.4	0		0	82.6	15.2	2.2
H3 reverse primer		97.7	2.3	0	0		84.8	4.3	10.9	0.0
H3 probe		81.8	4.5	4.5	9.1		0	95.7	4.3	0.0
fsH1 (2008–09)	196					196				
fsH1 forward primer		92.9	5.6	1.5	0		91.3	8.7	0	0
fsH1 reverse primer		99.0	1.0	0	0		95.4	4.6	0	0
fsH1 probe		91.3	8.7	0	0		88.3	11.2	0.5	0
09H1 (2018–19)	1,241					1,241				
09H1 forward primer		78.6	20.6	0.8	0		95.4	4.6	0	0
09H1 reverse primer		94.4	5.6	0	0		88.8	8.9	2.3	0
09H1 probe		0.2	91.9	7.8	0.1		1.8	42.3	54.2	1.7

a Based on sequence data obtained from U.S. isolates in the GISAID EpiFlu database.

## DISCUSSION

The Panther Fusion respiratory virus IVD assays (the FFABR, Paraflu, and AdV/hMPV/RV assays) are a modular approach to syndromic testing on a fully automated platform. This system and associated IVD assays have numerous benefits, such as exquisite sensitivity and onboard reagent stability. However, this system does not include a FluA virus subtyping assay, which is often part of a respiratory virus panel. With the Open Access system, LDTs can be used alongside IVD assays, with the same full automation. Our laboratory developed and compared two LDTs on the Fusion instrument for the subtyping of FluA virus: one assay using Hologic’s proprietary primers and probes from their Prodesse ProFAST+ assay and one using modified versions of primers and probes recommended by the WHO (7).

Both subtyping LDTs demonstrated clinical sensitivities comparable to that of the FFABR assay and were superior to many other commercial assays for FluA virus detection. The sensitivities of the cIAB, PFAST, RP, and Xpt assays in this sample set were 91%, 94%, 90%, and 85%, respectively, also by comparison to the FFABR assay. The sensitivities of 100% and 98.2% for the LDT-FAST and exWHO-FAST assays, respectively, were an impressive finding, because these samples had been subjected to repetitive freeze-thaw cycles for previous studies with the other assays. Another important point is that 97% of the H3 samples were taken from a previous prospective study involving consecutive specimens received in the lab during a 2-week period in the winter of 2015 (6). Analyses with such study populations predict assay sensitivities better than analyses of selected samples. During this 2-week period, the incidence of FluA virus was 25% and the subtype was exclusively H3. Indeed, the six previously untyped samples were collected during this time, and five of the six were typed as H3 by at least one of the typing LDTs. Initially, it appeared that there was one false-negative sample by both typing assays. However, repeat FFABR testing on the aliquot used for the subtyping assays was negative, indicating that either the virus titer was near the limit of detection for all three assays or the viral RNA had degraded.

We developed two assays due to an initial lack of certainty that our lab would continue to have access to the proprietary sequences from Hologic. Although both subtyping assays demonstrated sensitivities superior to those of many other commercial assays for FluA virus detection, the LDT-FAST assay was a little more sensitive than the exWHO-FAST assay. Interestingly, the FluA virus samples missed by the exWHO-FAST assay were from the 2-week period in 2015 in which the FluA virus population consisted primarily of clade 3C.2a viruses (10, 11). This clade had at least one mismatch with the exWHO-FAST forward primer and probe. Indeed, the WHO subsequently published an update to its recommended primers and probes for influenza virus detection, with an alternate target region for H3 viruses, for enhanced coverage (12). Despite the mismatches seen in the H3 primer and probe regions, the exWHO-FAST assay was still more sensitive for the H3 target than many other commercial assays. However, genetic drift does present a problem for any FluA virus typing system, because the HA gene is under the greatest environmental (immune) pressure to drift. It is necessary to watch diligently for frequent untypeable influenza virus-positive specimens, particularly if the *C_T_* values with the diagnostic FluA test are low, since assay modification may be needed. Fortunately, the risk associated with a false-negative typing result (FluA virus positive, subtype unknown) is minimal and is not an uncommon result with other IVD assays.

The value of including primers and probes for subtype fsH1, at an added cost of approximately $1, is certainly questionable. Our lab chose to include them because we did have two cases in 2018. Similarly, one may assert that viral clade determinations are needed, particularly since vaccine efficacy can differ with circulating viral clades, as seen with influenza virus A/H3 and FluB clades. However, the value in a clinical lab is currently minimal. Also, with regard to value, the benefit of running a subtyping assay concurrently versus sequentially with a FluA diagnostic assay would have to be addressed at each institution. Many of the experiments in this study were run concurrently, providing proof of concept that an LDT can be performed side by side with an IVD assay on the same sample and extract. It should also be noted that the current instrument software allows for only three assays from the same extract; however, Hologic is currently in the process of updating the system to allow for five assays per extract.

Besides the exquisite sensitivity seen with these FAST assays, the automation associated with the Fusion system has numerous other benefits. First, this is a fully automated process from the placement of the sample on the instrument to the reporting of results, which is certainly an improvement and reduces labor needs significantly. The instrument is designed for high-throughput testing with on-demand testing capabilities, and it complements Hologic’s modular approach to syndromic respiratory disease testing. Last, the system has reagent and consumable tracking with advance warning when more is needed.

In conclusion, both FluA virus typing assays were successfully adapted to run on the Panther Fusion instrument, with sensitivities comparable to that of the FFABR assay, providing a valuable complement to the Panther Fusion respiratory menu. This study also demonstrated proof of concept that LDTs and IVD assays can be processed side by side with full automation from sample to answer, either from the same sample or from the same eluate.
